# Comparison of Microscopy and Alamar Blue Reduction in a Larval Based Assay for Schistosome Drug Screening

**DOI:** 10.1371/journal.pntd.0000795

**Published:** 2010-08-10

**Authors:** Nuha R. Mansour, Quentin D. Bickle

**Affiliations:** Department of Infectious and Tropical Diseases, London School of Hygiene and Tropical Medicine, London, United Kingdom; McGill University, Canada

## Abstract

**Background:**

In view of the current widespread use of and reliance on a single schistosomicide, praziquantel, there is a pressing need to discover and develop alternative drugs for schistosomiasis. One approach to this is to develop High Throughput *in vitro* whole organism screens (HTS) to identify hits amongst large compound libraries.

**Methodology/Principal Findings:**

We have been carrying out low throughput (24-well plate) *in vitro* testing based on microscopic evaluation of killing of *ex-vivo* adult *S. mansoni* worms using selected compound collections mainly provided through the WHO-TDR Helminth Drug Initiative. To increase throughput, we introduced a similar but higher throughput 96-well primary *in vitro* assay using the schistosomula stage which can be readily produced *in vitro* in large quantities. In addition to morphological readout of viability we have investigated using fluorometric determination of the reduction of Alamar blue (AB), a redox indicator of enzyme activity widely used in whole organism screening. A panel of 7 known schistosome active compounds including praziquantel, produced diverse effects on larval morphology within 3 days of culture although only two induced marked larval death within 7 days. The AB assay was very effective in detecting these lethal compounds but proved more inconsistent in detecting compounds which damaged but did not kill. The utility of the AB assay in detecting compounds which cause severe morbidity and/or death of schistosomula was confirmed in testing a panel of compounds previously selected in library screening as having activity against the adult worms. Furthermore, in prospective library screening, the AB assay was able to detect all compounds which induced killing and also the majority of compounds designated as hits based on morphological changes.

**Conclusion:**

We conclude that an HTS combining AB readout and image-based analysis would provide an efficient and stringent primary assay for schistosome drug discovery.

## Author Summary

Only one drug, praziquantel, is widely available for treating schistosomiasis, a disease affecting an estimated 200 million people. Because of increasing usage there is concern about development of praziquantel drug resistance and a perceived need to develop new schistosomicides. Possible sources of these are large collections of compounds held by pharmaceutical companies and academic institutions. Anti-schistosome activity can be detected *in vitro* by visually assessing damage to cultured adult schistosome worms, but these are large and are recovered from mice which somewhat limits screening throughput. By contrast, schistosomula can be produced *in vitro* and used for screening in microwell plates, thus allowing medium throughput screening. High throughput screening (HTS) would require automated readout of schistosomulicidal action rather than manual microscopy. Here we report on the use of Alamar blue (AB), a fluorescent indicator of cell viability which can be measured rapidly and automatically. The AB assay was readily able to detect compounds causing death or severe damage to the larvae but was less reliable than microscopy for more subtle morphological changes including those induced by some known schistosome drugs. It is concluded that an automated HTS would benefit from integrated use of both AB and automatic image-based morphology assays.

## Introduction

Schistosomiasis is the most important helminth infection and the second most important parasitic disease next to malaria. It continues to spread in parts of the world due to water management and irrigation projects [1]. The major current strategy for control of schistosomiasis is chemotherapy and significant reductions in prevalence of infection and of severe disease have been achieved in several parts of the world e.g. Central and South America, North Africa and China [2]. A common approach is to treat all school-aged children in areas where the prevalence of schistosomiasis is over 10% and the whole community when the prevalence is over 50%. Such control has now been extended to several African countries through the Schistosomiasis Control Programme funded by the Bill and Melinda Gates Foundation and this has led to significant reductions in the prevalence, intensity and morbidity of infection [3]. However, chemotherapy does not interrupt transmission and so for this morbidity reduction to be maintained repeated periodic treatment will need to be continued for the foreseeable future.

Since its introduction in the 1980s praziquantel (Biltricide®) has been the mainstay of control programmes and it is now the only drug being used for mass treatment of schistosomiasis. It is an effective drug with broad spectrum activity against all five human schistosome species, low toxicity, few side-effects, simple administration and currently low cost. In mass treatment campaigns at a dose of 40 mg/kg praziquantel usually results in parasitological cure rates of around 70% and egg count reduction rates of more than 90% [4]. The marked increase in use of, and reliance on, repeated treatments with praziquantel has raised concerns about the possible emergence of drug resistance which, if it were to occur, would leave us without an effective schistosomicide [5]. There have been sporadic reports of low efficacy and of treatment failure in individuals from different parts of the world but as yet no convincing evidence of development and selection of resistance in endemic areas. However, strains of schistosomes which have been isolated from drug treatment failures show lower susceptibility to praziquantel and strains with stable elevated tolerance to the drug can be selected in the laboratory [6].

Worries about reliance on one schistosome drug and the possibility of the emergence of drug resistance led to the establishment of the “Helminth Drug Initiative (HDI)” (http://apps.who.int/tdr/svc/research/lead-discovery-drugs/workplans/helminth-drug-initiative) by the WHO Special Programme for Research and Training in Tropical Diseases (TDR) for the discovery of new schistosomicides [7]. As part of the HDI, whole organism screening was established with the London School of Hygiene and Tropical Medicine chosen as one of the screening centers. The screen adopted involved culture of adult worms with test compounds for 5 days with activity being determined by microscopic examination of worm death [8]. The HDI was initially based on highly selected libraries and throughput was not then the major consideration. However, with the provision of larger compound sets we implemented a 96 well microplate primary screen using *in vitro* derived schistosomula which allows markedly higher throughput than the adult worm-based assay. A similar assay was recently described by Abdulla et al [9]. Such assays are limited by the need for manual microscopic reading and, in order to develop a High Throughput Screen (HTS), the assay needs to be automated [10]. Therefore, alongside testing the use of High Content Screening (HCS) to allow automatic image analysis, we have investigated the use of a plate-based assay using Alamar Blue (AB), a redox indicator of enzyme activity which has been successfully used for colorimetric or fluorometric determination of viability of a number of protozoan parasites in whole organism drug screening e.g. African trypanosomes and *Leishmania* [11]–[12]. MTT (3-[4,5-dimethylthiazol-2-yl]-2,5-diphenyl tetrazolium bromide) which is also an indicator of enzyme activity has previously been shown to be reduced by adult schistosomes *in vitro* and used as a measure of parasite viability [13]. AB has the advantage over MTT in that it is non-toxic and so can be used to monitor effects over time.

We report here our results on the development and validation of the AB assay compared with morphology-based (microscopic) assessment of compound activity. For this purpose we have tested: (i) Several compounds (referred to here as “Standards”) with proven activity against schistosomes *in vivo* either in humans (oxamniquine [OX] [14]; praziquantel [PZ] [15]; methyl-clonazepam [MCZ] [16]; oltipraz [OPZ] [17]), in sub-human primates (Ro15-5458 [Ro15] [18]) or at least in mice (dihydroartemisinin [DHA] [19] [the analogue, artemether, has also been shown to be active in humans [20]]; clonazepam [CZ] [21]). In addition to their proven efficacy *in vivo* these Standard compounds were chosen because we had shown them to have a range of effects and speed of action against adult worms and schistosomula *in vitro*. Thus, PZ, MCZ, CZ and OPZ readily kill adult worms *in vitro* and have IC50s of 0.36, 0.37, 1.42, and 1.25 µg/ml, respectively (unpublished observations). However, OX, Ro15 and DHA have IC50s >10 µg/ml, the maximum concentration we use in our primary adult worm screening for WHO-TDR [8]; (ii) A panel of compounds found to be active or inactive by the adult worm assay during prior routine adult worm screening of compound libraries mostly supplied through the HDI (WHO-TDR); (iii) A subsample of compounds from a WHO-TDR library being screened for the first time.

## Materials and Methods

### Ethics statement

An ethics statement is not required for this work.

### Production of schistosomula

Cercariae of the Puerto Rican strain of *Schistosoma mansoni* [22] were shed in clean tap water from infected *Biomphalaria glabrata* snails exposed to direct illumination for 1 h. The cercariae were concentrated to 20 ml using an 8 µM filter (Sartorius CN, Scientific Laboratory Supplies, Ltd) on a concentration apparatus (Millipore) and cooled on ice for 30 min. The water was carefully removed from the pelleted cercariae and replaced by cold serum free medium 169 [23] (M169) supplemented with 300 U/ml Penicillin (Gibco, UK), 300 µg/ml Streptomycin (Gibco, UK), and 160 µg/mL Gentamicin (Sigma, UK) (Incomplete M169). Under sterile conditions, cercariae were mechanically transformed into schistosomula using the ‘Syringe Method’ [24]. The cercarial head and tail suspension was layered onto a sterile gradient of 50% and 60% Percoll (Sigma, UK) in M169 in 15 ml polystyrene tubes [25]. The tubes were spun for 10 min at 350× *g* at 4°C. After centrifugation each of the layers was carefully removed and the cercarial heads (schistosomula) recovered from the 60% layer, washed twice in 20–25 ml of Incomplete M169 by centrifuging at 400× *g* and 4°C for 2 min. The schistosomula were washed in M169 supplemented with 100 U/ml Penicillin, 100 µg/ml Streptomycin and 5% foetal calf serum (Sigma, UK) (Complete M169, cM169). They were then transferred into 6-well plates, incubated overnight at 37°C and 5% CO_2_ and checked for high viability (≤2% dead or damaged larvae) prior to use in screening.

### Drug sensitivity assay

Standard drugs: PZ, CZ and DHA were from Sigma, UK; MCZ and Ro15 were a kind gift from Dr H. Stohler (Hoffman-La Roche, Basle, Switzerland); OX was from Pfizer Ltd. Sandwich, UK; OPZ was provided by WHO-TDR, Geneva, Switzerland. These were dissolved in dimethylsulphoxide (DMSO) (Sigma, UK). Test compounds: these were from a number of different compound collections provided by WHO-TDR for routine screening using the adult worm assay. Since this is carried out at up to 10 µg/ml or (when compounds of known molarity are provided) at 12.5 µM, stock solutions of 10 mg/ml or 12.5 mM in DMSO were used. Mid-dilutions were performed as necessary in 100% DMSO and 1 µl added to 100 µl/well of cM169 in 96 well plates (Nunc, UK). Finally, 100 schistosomula were added in 100 µl of cM169 to each well. Negative control wells contained schistosomula cultured in either cM169 medium or 0.5% DMSO in cM169. The final concentration of 0.5% DMSO did not affect the larvae within the culture periods used.

Experiments with standard drugs were carried out in triplicate wells. The compounds were tested at 10 µg/ml (i.e. 28, 32, 30, 44, 32, 38, 35 µM for OX, PZ, MCZ, OPZ, CZ, Ro15 and DHA, respectively) or 1 µg/ml (i.e. 2.8–4.4 µM).

### Microscopic assessment of schistosomula viability

Viability of schistosomula was assessed on days 1, 3, 5 and 7 and in some experiments on day 14 using an inverted microscope (Leitz Diavert Wetzlar, Germany). Drug effects were determined by recording all schistosomula in each well as unaffected, dead (immotile, often showing a characteristic uniform shape and granular appearance) or morphologically damaged (showing a range of altered shapes, granularity and/or blebbing but still with some motility).

### Alamar blue assay

20 µl of Alamar Blue (AbD Serotec, UK) was added to each well and the plates incubated at 37°C and 5% CO_2_ for 24 h unless otherwise stated. The fluorescence intensity was measured using a Spectramax Gemini plate reader (Molecular Devices) using an excitation wavelength of 530 nm and an emission wavelength of 580 nm.

### Statistical analysis

Prism 4 (GraphPad Software, Inc) was used for graph drawing and statistical analysis. Student' t test was used to determine the significance of differences between mean values.

## Results

### Alamar blue reduction by *S. mansoni* schistosomula

**Figure 1:**
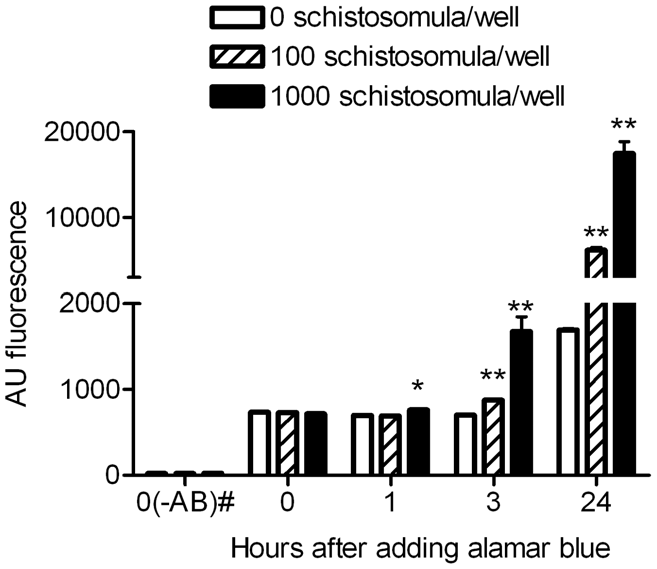
Alamar blue fluorescence development following culture with schistosomula. Wells contained 0, 100 (500/ml) or 1000 (5000/ml) schistosomula. # Wells containing schistosomula but no Alamar blue at time 0 (these values did not alter with time in culture). * P<0.01, ** P<0.0001 relative to wells with no schistosomula added. The mean ± standard deviation of fluorescence, expressed in arbitrary units (AU), is based on 5 replicate wells.

An initial experiment showed that AB could readily detect metabolic activity during schistosomula culture. As shown in Figure 1 there was induction of significant (P<0.01) fluorescence as early as 1 h following incubation with 1000 schistosomula (5000/ml). By 3 hrs significant levels of fluorescence were seen with both 100 and 1000 schistosomula and this was significantly enhanced (P<0.001) at 24 h (4 and 25 fold higher fluorescence levels, respectively, for 100 and 1000 schistosomula compared with control without parasites). At each time point 1000 schistosomula induced significantly greater fluorescence than 100 (at 1 and 3 h P<0.01; at 24 h P<0.0001) but use of such high numbers of larvae for screening would greatly reduce possible throughput. Based on such data, future experiments with AB used 100 schistosomula per well read at 24 h or later. In preliminary experiments we showed that OPZ, which kills schistosomula *in vitro*, inhibited this AB conversion and so we were encouraged to test the assay further.

### Comparison of Microscopy-based and Alamar blue assays using known schistosome active compounds (standards)

#### Microscopy-based assay (morphological observations)

**Figure 2:**
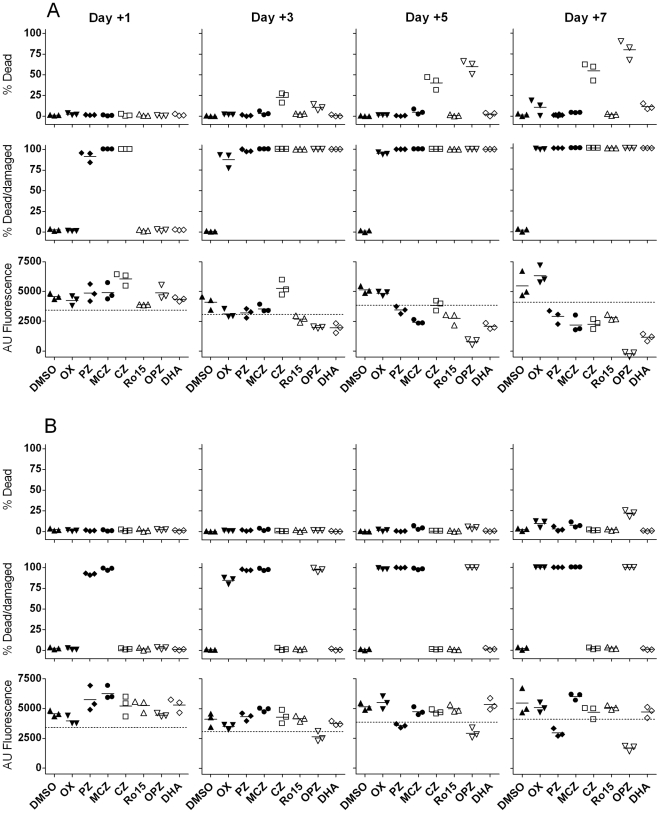
Comparison of microscopy and Alamar blue to assess activity of known anti-schistosome drugs. Replicate 96-well microtitre plates were set up containing DMSO at 0.5% and either 10 µg/ml (Figure A) or 1 µg/ml (Figure B) of Oxamniquine (OX), Praziquantel (PZ), Meclonazepam (MCZ), Clonazepam (CZ), Ro15-5458 (Ro15), Oltipraz (OPZ) or Dihydroartemisisin (DHA). 100 schistosomula were added per well. Triplicate wells were set up for each treatment. The % dead or damaged schistosomula were counted on each of days 1, 3, 5 and 7. Alamar blue was added on these days and fluorescence read in Arbitrary Units (AU) 24 h later. Mean fluorescence values from wells containing AB and cM169 without parasites were subtracted from the values for test wells. The results for each of the triplicate wells are shown in the graphs with the horizontal bars representing the mean values. The dotted horizontal line on each AB graph corresponds to an AU value 25% lower than the mean for the triplicate DMSO controls. Values for medium alone controls were very comparable to the DMSO values (data not shown).

Using 10 µg/ml (Figure 2A) only CZ and OPZ caused elevated larval killing over the time course, increasing from day 3 to day 7 when 54% and 80% killing was observed, respectively. However, all of the Standards induced marked morphological changes (damage) to the larvae. By day 1 90–100% of larvae treated with PZ, MCZ or CZ showed damage or death and by day 3 all Standards induced >75% damage or death. By comparison 1 µg/ml (Figure 2B) induced very little larval death at any time point but OX, PZ, MCZ and OPZ caused >75% damage on days 3–7 whereas CZ, Ro15 and DHA failed to induce morphological changes at any time-point. Such experiments with the Standard drugs have been reproduced many times and in view of this and our experience using the larval assay in primary compound screening we routinely read the microscopy assay at day 3 and define a hit as causing ≥70% damage or death.

#### Alamar blue assay

Graphs of the AB results for the Standards are also shown in Figure 2. For comparative purposes the dotted horizontal lines on these graphs represent AB values 25% lower than the mean value for the DMSO control wells. The choice of 25% inhibition is based on overview of all of the results from these studies and represents a value which appears to be a useful cut-off for establishing hits based on drug-induced inhibition of metabolism (i.e. lower AB fluorescence values).

Using 10 µg/ml of the Standards (Figure 2A) there was little effect by day 1. By day 3 Ro15, OPZ and DHA had inhibited AB values by >25% compared with controls. On day 5 OPZ and DHA showed >50% inhibition, MCZ and Ro15 were close to 50% and PZ and CZ were close to the 25% level although some wells showed <25% inhibition. By day 7 all of the drugs apart from OX resulted in AB inhibition of >25%. When tested at µg/ml (Figure 2B) only PZ and OPZ showed inhibition >25% by day 7. Extending the culture period to 14 days and also increasing the AB reading times to +48, +72 and +96 h did not enhance the discrimination between controls and these Standards (data not shown) and so culture for 7 days was considered to be the optimal duration for this assay with >25% AB inhibition as the cut-off.

### Comparison of Microscopy-based and Alamar blue assays using compounds active against the adult worms *in vitro*

We next tested the larval assay against a random collection of 33 compounds which had proved to be active *in vitro* at 10 µg/ml against adult schistosomes in our standard assay [8] (i.e. “Adult +ve”) along with 30 randomly selected adult negative compounds (“Adult −ve”).

Microscopy-based assay (morphological observations).

**Figure 3:**
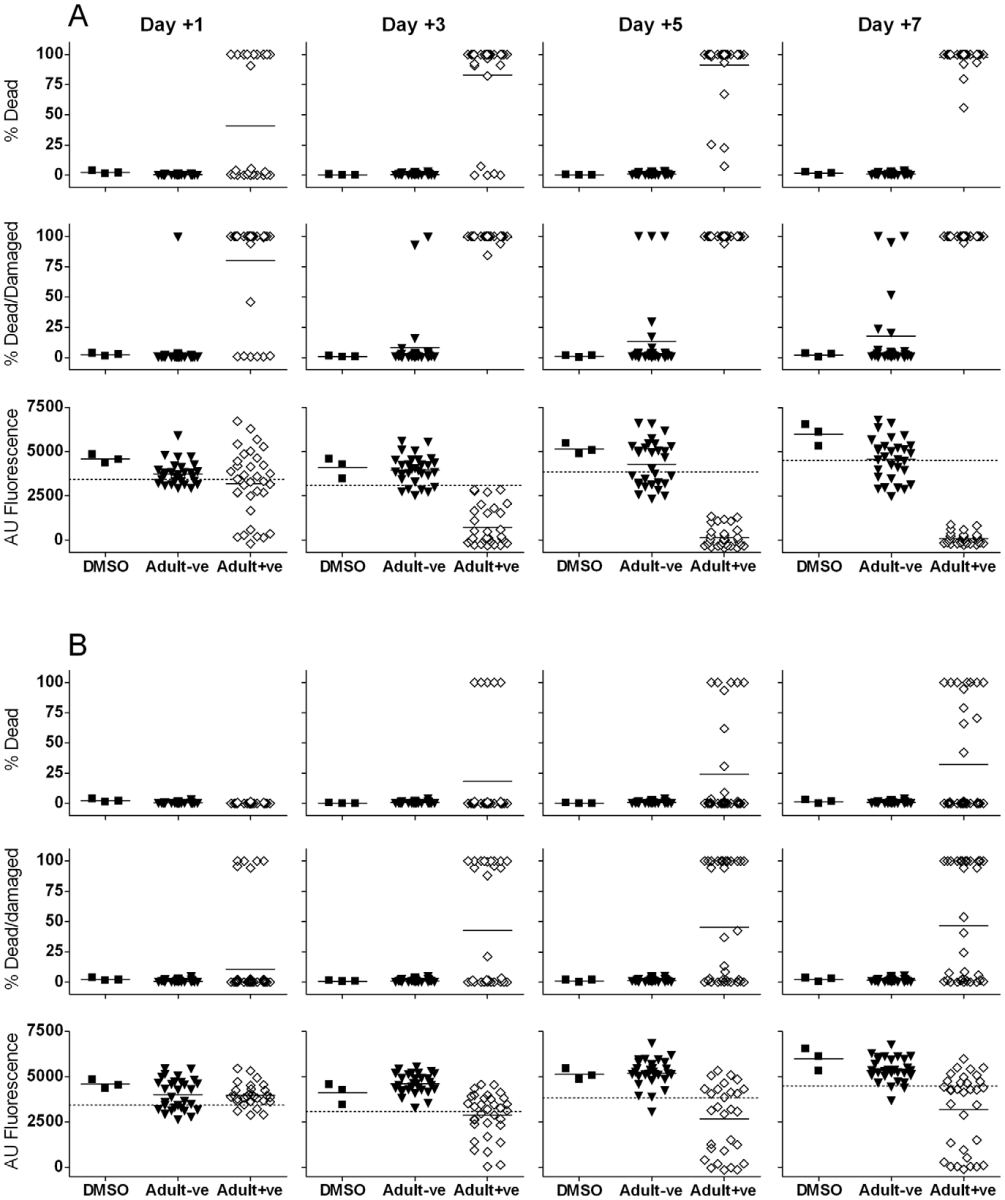
Comparison of microscopy and Alamar blue to assess activity of compounds previously screened as active or inactive against adult schistosomes *in vitro*. See Legend to Figure 2 for most details. A: 10 µg/ml, B: 1 µg/ml. “Adult +ves”: 33 compounds previously found to be lethal to adult worms *in vitro*. “Adult −ves”: 30 compounds found to be inactive against adult worms *in vitro*.

Figure 3A shows the results using 10 µg/ml of these compounds. 10/33 (30%) of Adult +ves had killed all or nearly all the larvae by day 1 and 80% were classified as hits (≥70% damaged or dead). By day 3, 28/33 (85%) had induced killing of most larvae and all were detected as hits. Larval death increased at days 5 and 7 at which time all of the compounds caused >50% death and all but 4 induced 100% parasite death.

Using 1 µg/ml (Figure 3B) only 14/33 (42%) would have been classified as hits on morphological grounds on days 3, 5 and 7.

None of the Adult −ves induced significant parasite death at either drug concentration and by day 3 only two were classed as hits based on morphological damage when tested at 10 µg/ml, giving a false positive rate of ∼7% (NB “false positive” is used here to define activity relative to the adult assay. The latter is taken as the reference because it is used as the secondary and definitive screen for hit progression. Activity in the larval but not the adult screen may be due to higher sensitivity of the schistosomula or to larval specific action). At 1 µg/ml there were no false positives amongst the Adult −ves.

#### Alamar blue assay

Figure 3A shows the results using 10 µg/ml. Following addition of AB on day 1, all 10 of the Adult +ve compounds which had resulted in visible larval death at this time induced AB inhibition of ≥25%. Overall the assay detected 16/33 (48%) as hits (≥25% AB inhibition) compared with the 80% detected visually. By day 3 all of the Adult +ves had induced AB inhibition of ≥25% although some values were only just below the 25% level. By days 5 and 7 all Adult +ves caused inhibition of AB reduction of >50% compared with controls. However, 12/30 of the Adult −ves also gave Alamar blue inhibition of ≥25% at day 7 (i.e. a false positive rate of 40%). The range of AB values observed with the Adult −ves reflects compound-specific effects rather than random variation between wells e.g. numbers of larvae per well, since when compounds were tested in duplicate there was a highly significant correlation between paired observations (P<0.0001) (data not shown).

Using 1 µg/ml (Figure 3B) 22/33 (67%) Adult +ves gave AB inhibition of ≥25% at day 7 and there was only one false positive (AB inhibition ≥25%) amongst the Adult −ves (3%).

Taking the results with the Standards and the Adult +ves together, the 3 day morphology-based assay detected all of these as hits at 10 µg/ml whereas testing at 1 µg/ml resulted in a failure to detect 3/7 of the Standards and 58% of the Adult +ves. The day 7 AB assay was also effective in detecting the Standards (apart from OX) and the Adult +ves at 10 µg/ml but gave a high false positive rate (40%) with the Adult −ves. At 1 µg/ml the AB assay failed to detect 5/7 of the Standards.

### Comparison of the Microscopy-based and AB assays during routine primary screening

**Figure 4:**
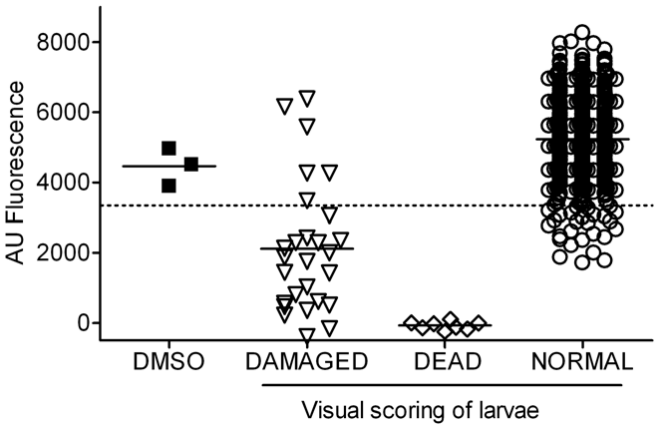
Comparison of microscopy and Alamar blue assays during routine screening. See Legend to Figure 2 for most details. Results are shown for the screening of 558 compounds (i) by microscopy at day 3 of culture, larvae being reported as DEAD, morphologically DAMAGED or NORMAL and (ii) by addition of Alamar blue on day 7 and reading 24 h later.

In the following experiment we compared the 3 day microscopy and 7 day AB assays during primary screening of 558 compounds. These were from a compound library provided by WHO-TDR at known molarity in which circumstance primary screens are run at 12.5 µM. As shown in Figure 4, the visual assay detected 8 compounds lethal to the larvae and 27 designated as hits based on damage i.e. a total of 35 “hits” (∼6% hit rate). The AB assay readily detected all 8 of the drugs causing larval death and 21/27 (78%) of the morphologically damaged hits. In addition there were 27/558 compounds causing ≥25% inhibition in the AB assay which were not detected as hits by morphology. Overall, therefore, 56/558 compounds induced AB inhibition of ≥25% i.e. a hit rate of 10%.

**Figure 5:**
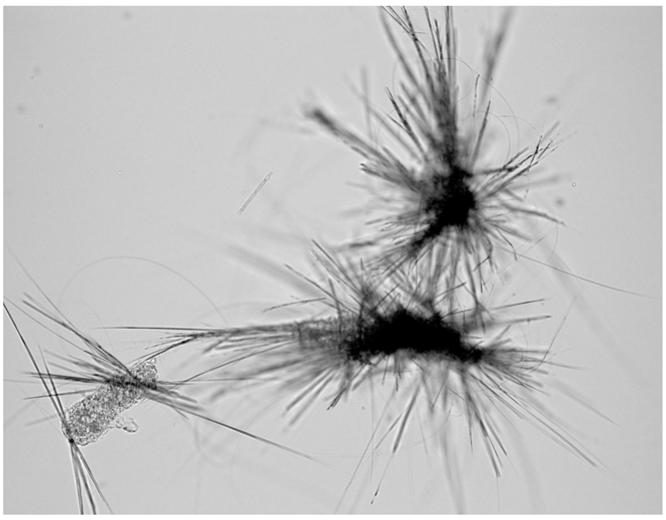
“Crystal-like” formation on schistosomula. An example of crystal-like deposit from one of the wells containing schistosomula categorized as DAMAGED from Figure 4. (Magnification ×100). Image captured using a Leitz DMRB microscope (Leica, Germany) with attached Leica DFC 420 digital camera controlled by Leica Application Suite version 3.1 software (Leica Microsystems [Switzerland] Ltd.).

The 35 visual hits were tested in the secondary *ex-vivo* adult assay and 5 were shown to be positive. All of these were in the group of 8 compounds which had resulted in schistosomula death by day 3 and which also caused marked inhibition of AB fluorescence at day 7. Of the 35 visual hits 6 caused crystal formation/drug deposition in or on the parasites and in some cases this seems to pierce the larvae e.g. Figure 5. Such effects are not uncommon and assessment of the morphological condition of the larvae can be difficult in such cultures. None of the 6 were hits in the adult assay and two of them gave the highest AB AU fluorescence values in Figure 4 indicating that the larvae were still highly metabolically active.

## Discussion

Abdulla et al [9] recently described a medium throughput drug screen based on microscopically determined morphological changes to schistosomula. It was reported that attempts to quantitate cell death in schistosomula using nuclear dyes did not correlate with observed morphological effects seen under bright field microscopy. Our experience supports the conclusion that morphological changes to schistosomula represent a rapid and sensitive means of detecting anti-schistosome activity. An assay based on inhibition of AB reduction was also very effective in detecting severely damaged and dead schistosomula and with a sufficiently long culture period (7 days) was able to detect most of the known schistosome active compounds tested.

The microscopy-based morphological assay described by Abdulla et al [9] involved a primary schistosomula screen at 7 days, a secondary similar screen at 24 h to detect fast-acting compounds and then a tertiary screen using *ex vivo* adults read at day 1–4. Priority for progression to the adult screen was based on activity against schistosomula at 24 h. Based on initial screening of two commercially obtained compound collections it was concluded that the hit rates of 25 and 15% when screening was at 10 and 5 µM were too high whereas the hit rate of 10% at 1 µM was acceptable. In our experiments with a range of known schistosome active drugs (Standards) we found that the microscopy-based larval screen described here, which is standardly read at day 3, detected all 7 compounds as hits when tested at 10 µg/ml (i.e. 28–44 µM) but at 1 µg/ml (i.e. 2.8–4.4 µM) CZ, Ro15 and DHA were not detected even at 7 days. The failure to detect Ro15 and DHA at 1 µg/ml may be seen as a failure of the assay but in practice these two compounds would not have been detected in our secondary adult worm screen since they are inactive against the adult worms *in vitro* at 10 µg/ml. In our testing of a collection of 33 compounds originally detected as hits by primary screening against adult worms, all were detected at 10 µg/ml, but at 1 µg/ml 58% were not detected even when cultured up to 7 days. We conclude that testing at 1 µg/ml (estimated at ∼3 µM) missed an unacceptable percentage of the Adult +ves. As mentioned above the choice of 1 µM in the earlier report [9] was an empirical one based on achieving an acceptable hit rate (∼10%). In the present studies we found that a selection of Adult −ve compounds from the collections which yielded the Adult +ves gave a low false positive rate of ∼7% at 10 µg/ml. Since these Adult −ves were randomly selected from the libraries this value of 7% represents the hit rate which would be expected from these collections using the larval assay at 10 µg/ml. However, in our prospective screening of new collections of compounds using the schistosomula primary screen at 10 µg/ml hit rates up to 16% have been obtained whilst with compounds tested at 12.5 µM we have found an average of 6% hits. Given the variation in hit rates seen with different libraries, the rational approach is to determine a concentration that gives an acceptable hit rate for progression to secondary screening [9].

It is interesting that all of the compounds we tested here which had been found to be active in the adult worm screening of WHO-TDR collections proved to be highly active against the schistosomula as judged visually. Thus of 38 such adult actives tested all but 5 had killed >75% of larvae and all had caused ≥70% morphological damage by day 3. By contrast the Standards produced more diverse effects, readily detectable by morphological damage but, with the exception of OPZ, not resulting in high levels of parasite death up to a week in culture.

AB fluorescence was found to be a reliable indicator of schistosomula viability and the AB assay was able to detect severely damaging and lethal effects caused by test compounds. However, it was much less effective in detecting the more subtle morphological effects such as those caused by most of the Standards. For example, OPZ which kills around 75% of larvae by day 7 in culture at 10 µg/ml was reliably detected as causing >25% inhibition of AB conversion from day 3 onwards, reaching 100% inhibition by day 7. On the other hand PZ, MCZ, CZ and Ro15 which damage all but kill few schistosomula within 7 days had more modest effects on AB conversion although, if cultured up to 7 days, ≥25% inhibition was generally observed. However, having run these Standards as controls in many screens we have noted that occasionally these four compounds fail to show a ≥25% inhibition and so would not be detected as hits. Altering test compound concentrations, culture duration and interval to AB reading did not lead to a protocol which improved this. We conclude that relative to microscopy the AB assay would occasionally miss detecting PZ, Ro11, CZ and Ro15 and would not detect OX. It is, however, worth noting that neither Ro15 nor OX are active in the adult *in vitro* visual assay and so would not be taken forward as hits following secondary screening.

Comparison of the morphology and Alamar blue assays against collections of compounds previously shown to be positive or negative against adult worms *in vitro* showed that both assays were 100% sensitive in detecting the Adult +ves at 10 µg/ml. However, whereas the microscopy assay was positive for only ∼7% of Adult −ve compounds from these source libraries, the AB assay produced a wide range of values with 40% of these falling below the cut-off for hits in this assay. When the Adult −ves were tested at 1 µg/ml the AB assay showed only 3% as hits and also showed a somewhat higher sensitivity in detecting Adult +ves (67%) than the morphology assay (42%). Since the Adult −ve compounds were randomly chosen from compound collections sent for testing, the 40% and 3% “hit” rates with 10 and 1 µg/ml, respectively, reflect the hit rates which would be obtained if the AB assay were used for primary screening of these collections. As discussed above the optimal concentration for a particular library would need to be chosen empirically. So, for AB screening of this particular compound collection the appropriate concentration would be somewhere between 10 and 1 µg/ml in order to yield a manageable hit rate for progression to the adult worm secondary screen. Notably, in this retrospective testing of the Adult +ves at 1 or 10 µg/ml, all wells, apart from 2 on Day 1, where there was larval death (range 10–100% cf controls 0.2–2%), the AB values were >25% lower than the controls and in most instances >50% lower. Similarly, in the prospective testing at 12.5 µM (Figure 4) all wells in which larval death was recorded (37–100% death) were readily detected by the AB assay.

In conclusion the AB assay has proved to be very effective in detecting compounds which have severe effects on the larvae resulting in a proportion of the larvae dying. The assay is also very suitable for HTS automation and is valuable when there is crystal formation on the parasites which obscures accurate observation. However, it showed somewhat lower sensitivity and reliability compared with manual visualization in detecting the more subtle damage caused by some known anti-schistosome compounds including praziquantel. Therefore, we envisage an HTS may usefully incorporate both AB and image-based analysis and are currently investigating if automatic HCS image-based analysis can be developed which has comparable sensitivity to the manual visualization assay.
